# Comparison of Ultrasonic Non-Contact Air-Coupled Techniques for Characterization of Impact-Type Defects in Pultruded GFRP Composites

**DOI:** 10.3390/ma14051058

**Published:** 2021-02-24

**Authors:** Aadhik Asokkumar, Elena Jasiūnienė, Renaldas Raišutis, Rymantas Jonas Kažys

**Affiliations:** 1 K. Baršauskas Ultrasound Research Institute, Kaunas University of Technology, K. Baršausko St. 59, LT, 51423 Kaunas, Lithuania; elena.jasiuniene@ktu.lt (E.J.); renaldas.raisutis@ktu.lt (R.R.); rymantas.kazys@ktu.lt (R.J.K.); 2 Department of Electronics Engineering, Kaunas University of Technology, Studentų St. 48, LT, 51367 Kaunas, Lithuania; 3 Department of Electrical Power Systems, Kaunas University of Technology, Studentų St. 48, LT, 51367 Kaunas, Lithuania

**Keywords:** ultrasound, guided wave testing, ultrasonic-guided wave tomography, Lamb waves, tomographic reconstruction, pultruded GFRP, nondestructive testing (NDT), air-coupled

## Abstract

This article compares different air-coupled ultrasonic testing methods to characterize impact-type defects in a pultruded quasi-isotropic glass fiber-reinforced plastic (GFRP) composite plate. Using the air-coupled transducers, comparisons among three methods were performed, namely, bulk-wave through transmission, single-side access using guided waves, and ultrasonic-guided wave tomography. The air coupled through transmission technique can determine the size and shape of impact-type defects with a higher resolution, but with the consequence of time consumption and, more importantly, the necessity of access to both sides of the sample. The guided wave technique on the other hand, allows a single-side inspection and is relatively fast. It can be used to determine the size of the defect using ultrasonic B-scan, but the exact shape of the defect will be compromised. Thus, in this article, to determine the shape of the defect, application of the parallel beam tomographic reconstruction technique using guided Lamb waves is demonstrated. Furthermore, a numerical finite element simulation was performed to study the effects of guided wave propagation in the composite sample and interaction with the internal defect. Lastly, the results from the experiments of different techniques were compared according to possibilities of defect sizing and determination of its shape.

## 1. Introduction

In the modern world, composite materials are dominating in the aerospace industry with almost 50% of aircraft made from composite materials [1]. Composite materials are also being used more and more in wind turbines [2] and other structures due to their high strength-to-weight ratio and resistance to corrosive damage [3]. Using pultrusion of fiber-reinforced plastics allows producing long sections of a composite material with a uniform cross-section at a relatively low cost. In the aerospace industry, unidirectional pultruded glass fiber-reinforced plastic (GFRP) composites demonstrating high stiffness in one direction are used in parts such as spar caps, panel stiffeners, and longerons [4]. When such GFPR composites are exposed to bird strikes, hailstorms, etc., some researchers studied the effect of impact-type defects in composites [5,6,7,8]; to summarize, the size and shape of these defects can vary depending on the orientation of the glass fiber matrix and the ratio of glass fiber to volume matrix which can lead to micro fiber cracking, delamination, internal matrix fiber cracking etc. In such cases, the severity of the damage cannot be accessed visually and, in order to inspect such impact-type defects, nondestructive testing needs to be used.

Ultrasonic testing (UT) is one of the most popular techniques for the inspection of different structures nondestructively. Conventional ultrasonic testing requires a liquid acoustic coupling for inspection. However, wetting of composites with coupling liquids is undesirable and very often is not even allowed because it may cause more damage to the structure due to ingress of liquids. Therefore, air-coupled ultrasonic testing is favorable for inspection of the composite structures. UT comprises a wide variety of techniques. According to the distance covered during the inspection, it can be separated into bulk-wave testing and guided wave testing.

A through transmission technique exploiting bulk waves is traditionally used extensively for nondestructive testing (NDT). However, in the case of the through transmission technique using an ultrasonic bulk wave, inspection can be performed only at the insonified area under the ultrasonic probe. Therefore, in such a way, it is possible to obtain high-resolution C-scans in a localized region, but access to both sides of the inspected structures is needed. Such an approach was previously applied for composite inspections using the through transmission technique [9,10,11]. Studies [12,13,14,15] showed that air-coupled testing shows promising results in the detection of internal defects in composites, and techniques such as air-coupled imaging using the pulse compression method [16,17] can be used for improving the signal-to-noise ratio.

In the case of ultrasonic guided wave inspection, the Lamb waves travel inside a plate-like structure with a low loss of energy. Losses depend on the attenuation of ultrasonic waves due to material and radiation of the leaky waves. Usually, the losses in materials are frequency-dependent and increase with frequency. The losses in plastic and composite materials are higher than those in metals. For example, in plastics with high attenuation losses such as polypropylene and polyvinylidene fluoride (PVDF), they are between 2 dB/cm at 300 kHz and 5 dB/cm at 500 kHz [18,19]. In the case of noncontact inspection, one of the major losses to be considered constitutes the losses related to the large acoustic impedance mismatch between the guided wave medium and the air. Losses due to radiation of leaky waves may be very significant when the structure is immersed in water; however, in the case of air, the environmental losses are much lower. However, the frequency dependence of attenuation losses of guided waves is much more complex and depends not only on the radiation of leaky waves, but also on guided wave modes. For example, it was shown that, in the case of the slow subsonic antisymmetric A0 Lamb wave mode, when a leaky wave is not radiated, the attenuation coefficient can still reach 2 dB/cm even at low 44 kHz frequency [20].

Nevertheless, guided waves can travel long distances in the inspected structures and are suitable for long-range inspections. Depending on the material, the complexity of geometry, and the wavelength of the excited guided wave, distances up to a few meters can be covered during an inspection. Guided wave testing showed promising results for testing of composite specimens [21,22].

The guided wave technique discussed above allows the detection of delamination and impact-type defects but does not precisely reveal the shape and internal structure of the damaged region. Such information may be required when, for example, structure after a fatal event is investigated. In such a case, ultrasonic tomography can be applied. Tomographic reconstruction of an object is based on an algorithm of imaging by obtaining projections at a series of angles. The motivation of ultrasonic tomography using air-coupled transducers dates even back to the 1990s [23,24], where simple metallic aluminum plates and 16-ply composite specimens were investigated. Furthermore, a study from 2014 [25] was performed on isotropic polyvinyl chloride (PVC) polymer to identify volumetric voids and planar inclusions, showing promising results using air-coupled tomography. There were studies on ultrasonic tomography using contact transducers [26,27,28,29] which showed promising results with faster solutions; however, the biggest downside is that, for these contact techniques, the transducers are fixed at a permanent position, which offers a structural health monitoring solution, but is not suitable for in situ inspections. Another recent study was performed [30] using laser excitation; the air-coupled approach was used for isotropic materials and various tomographic reconstruction algorithms were discussed.

Considering all these studies regarding tomography imaging, this research focuses on the air-coupled excitation and reception of ultrasonic waves in a pultruded composite GFRP specimen.

The objective of this article was to compare different air-coupled ultrasonic techniques for testing of the pultruded type GFRP composite with impact damage. This kind of comparison of different air-coupled testing methods was not performed in other studies. Ultrasonic air-coupled techniques were compared with contact and immersion techniques for evaluation of bonding quality. A comparison of several techniques using ultrasonic Lamb waves for a 2 mm aluminum sample was performed in [31], and a review of different air-coupled testing techniques was presented in [32] with a different sample for each technique. Thus, the novelty of this research is in its comparison of the following air-coupled ultrasonic methods for the inspection of the pultruded GFRP composite with impact-type defects:

- Air-coupled through transmission method using 300 kHz single-element unfocused transducers and 485 kHz composite focused transducers;
- Air-coupled guided waves using 300 kHz single-element unfocused transducers;
- Guided wave tomography using 300 kHz single-element unfocused transducers.

## 2. The Specimen Used for the Study

**Figure 1:**
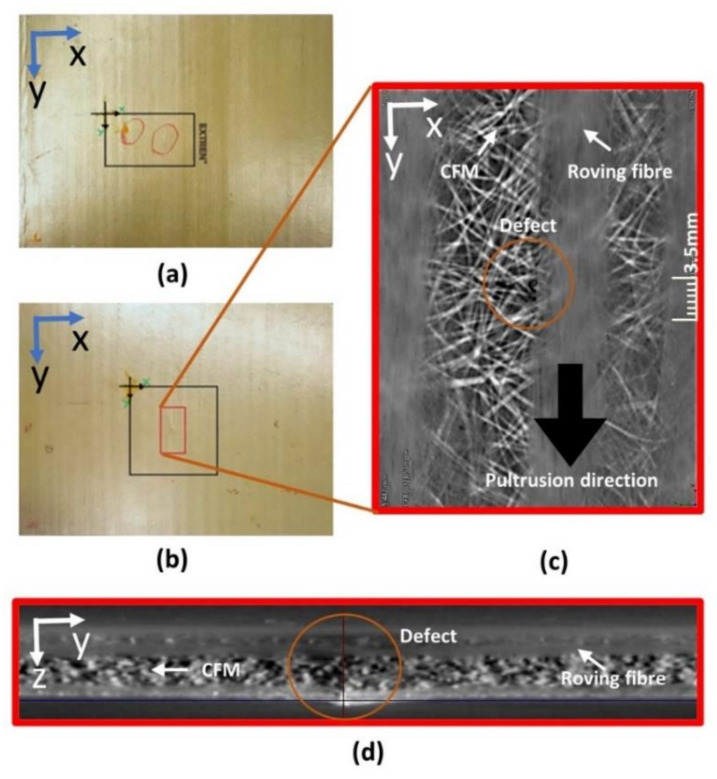
Pultruded glass fiber-reinforced plastic (GFRP) composite specimen with 8 J impact damage (**a**) and 12 J impact damage (**b**). The red area indicates the location of the impact damage. Cross-section of the X-ray computed tomography (CT) scan of sample with 12 J impact defect in *x*–*y* plane (**c**) and *y*–*z* plane (**d**).

The pultruded GFRP composite specimens made of E-glass fibers and vinyl ester resin with artificial impact-type defects were provided by the University of Warwick (Coventry, UK) where previous inspections with capacitive air-coupled transducers were performed using the pulse-echo technique. Two samples of pultruded GFRP composite plates with 8 J (Figure 1a) and 12 J (Figure 1b) impact defects were investigated in this study.

To visualize the internal structure of the GFRP specimen, X-ray computed tomography inspection was performed on the 12 J impact specimen. The X-ray computed tomography was performed using a RayScan 250E X-ray computed tomography device (RayScan Technologies GmbH, Meersburg, Germany). For that, a 10–230 kV X-ray source with the following parameters was used: 200 kV voltage, 200 µA current; 999 ms integration time. A total of 3600 projections with a resolution of 42 µm were measured. Data were averaged three times, and the results are shown in Figure 1c,d at different cross-sections.

From the cross-section of the X-ray CT three-dimensional (3D) data, shown in Figure 1d, the arrangement of the GFRP laminate was determined. It consisted of unidirectional roving fibers made of glass sandwiched between a randomly distributed continuous fiber matrix (CFM) made of plastic. Such a GFRP was discussed in [4,33,34,35]. The roving fibers were found to be flat and present on top of the plate, and these roving fibers created a waviness pattern, which can be observed in Figure 1a,b.

The provided GFRP samples were assumed to have isotropic elastic properties because the main part of the plates was made of CFM material. The dimensions of those plates were 305 × 241 × 3.2 mm^3^, and the elastic properties were not known. To generate guided waves, the elastic properties are essential to determine which mode is feasible to generate and how to generate a specific mode. The material of CFM was assumed to be made from vinyl ester matrix (*m*) and the glass fiber (*f*) was assumed to be made of E-glass; the material properties of these materials, as shown in Table 1, were considered according to [36]. The elastic properties of the GFRP plate were calculated to be Young’s modulus *E* = 10.5 GPa, Poisson’s coefficient *ν* = 0.36, and density *ρ* = 1342 kg/m^3^ using Equation (1).

(1) $$X=V_{f}X_{f}+V_{m}X_{m},$$

where *X_f_* is the elastic property of the fiber material, *X_m_* is the elastic property of the matrix material, *V_f_* is the volume fraction of 0.1 for glass fiber, and *V_m_* is the volume fraction of 0.9 for CFM. Here, the volume fractions were assumed on the basis of the X-ray results, as it can be seen that the majority of volume was from CFM. This assumption is verified in the next section.

## 3. Techniques for Investigation

**Figure 2:**
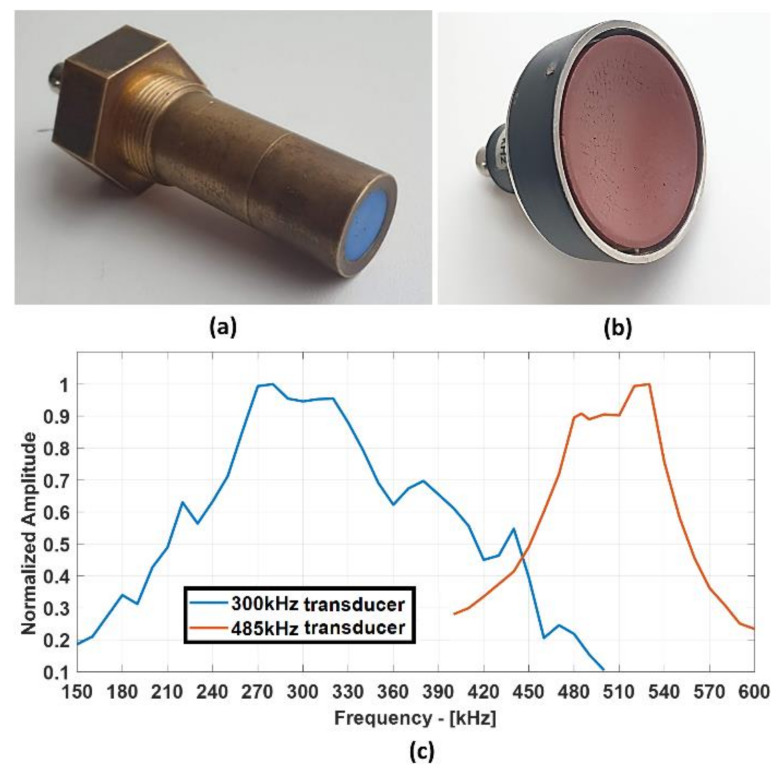
Air-coupled transducers (300 kHz unfocused transducer (**a**) and 485 kHz focused transducer (**b**)) and their frequency responses (**c**).

The GFRP specimen was investigated using the bulk-wave through transmission technique with contactless air-coupled non-focused and focused transducers to obtain the size and shape of the impact damage at high resolution. Two pairs of transducers manufactured at the Ultrasound Institute (UI) of Kaunas University of Technology (Kaunas, Lithuania) were used for the measurements. One pair consisted of single-element unfocused air-coupled transducers with 300 kHz operating frequency, whereas the other consisted of composite focused air-coupled transducers with 485 kHz operating frequency. The unfocused 300 kHz air-coupled transducers were used for both bulk-wave and guided wave investigations, while the focused air-coupled 485 kHz transducers were used only for the bulk-wave through transmission testing. The transducers used in this investigation are shown in Figure 2 along with their frequency responses. The active transducer diameter for the flat transducer was 14 mm and that for the focused transducer was 40 mm. The curvature of the focused transducer was 34 mm, and the diameter of the focal spot at −6 dB was measured to be 1 mm.

### 3.1. Bulk-Wave through Transmission Technique

**Figure 3:**
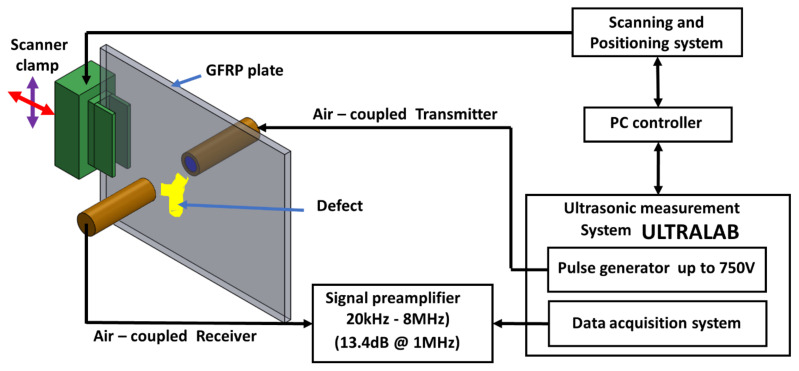
Setup for the air-coupled through transmission inspection.

The active diameter of the single-element 300 kHz air-coupled transducer was 14 mm. In the case of the bulk-wave through transmission technique, the flat transducers were placed at the calculated nearfield distance of 43 mm on both sides from the surface of the GFRP plate. In the case of the through transmission technique using the focused transducers, they were positioned at the distance of 78 mm with the GFRP plate in the middle. The bulk-wave through transmission setup is shown in Figure 3.

### 3.2. Guided Lamb Waves and Dispersion Curves

The air-coupled guided wave method is a three-step process, which is described as follows:

1. The air-coupled transmitter placed at a specific angle generates ultrasonic longitudinal waves (plane waves) in air;
2. The incident plane wave is transferred into the waveguide (plate-like sample) and causes constructive and suppressive interference, transforms, and propagates as guided Lamb waves;
3. Guided waves traveling inside the waveguide cause mechanical perturbation in the surrounding air medium, which generates leaky Lamb waves. The leaky Lamb waves are measured using the air-coupled receiver.

**Figure 4:**
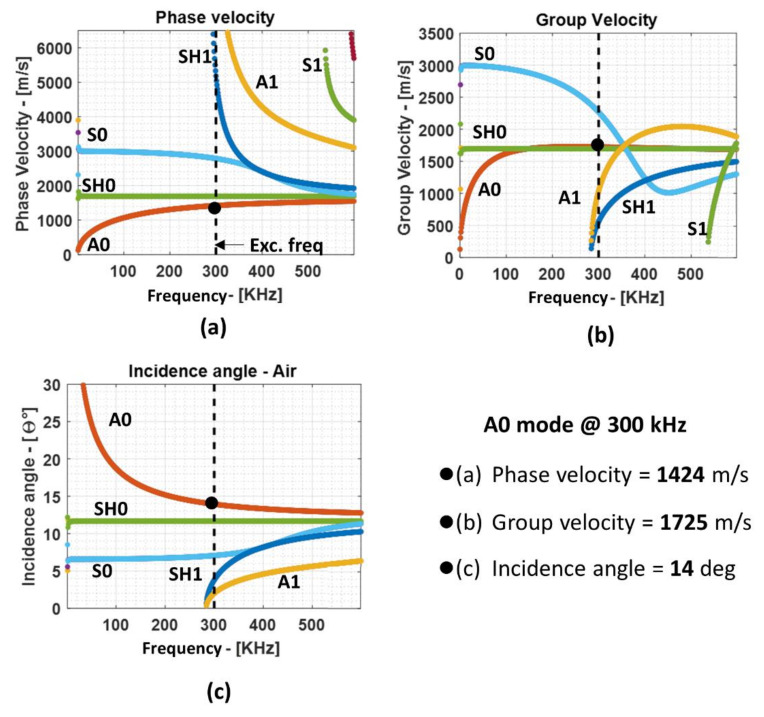
Phase velocity (**a**), group velocity (**b**), and the incidence angle plot (**c**) for the 3.2 mm GFRP composite plate.

In the guided wave technique, several modes can be excited at the same time at a specific frequency. This is critical because some specific modes are sensitive to the specific type of defects. Therefore, a selective guided wave mode excitation and reception are of interest in the case of guided wave inspection. To know the different types of modes propagating at different frequencies, dispersion curve plots are used. The dispersion relation was solved using the semi-analytical finite element (SAFE) method for the Lamb waves. The reader is referred to [37] for SAFE formulation, where it is described that this technique can be used not only for isotropic materials abut also for layered anisotropic materials after homogenization. The phase velocity, the group velocity dispersion curves, and the required incidence angle for each mode for the 3.2 mm GFRP plate are shown in Figure 4a–c, respectively.

The specific guided wave modes can be generated by changing the incidence angle *θ_i_*. The incidence angle can be calculated using Snell’s law, as shown in Equation (2).

(2) $$\theta_{i}=\arcsin\left(\frac{V_{air}}{V_{ph}}\right),$$

where *V_ph_* is the phase velocity mode in the GFRP plate from dispersion curves and *V_air_* is the velocity of sound in air.

From the dispersion curves, it is apparent that, in the waveguide, several modes can be generated at the same time at higher frequencies. Hence, lower frequencies are usually preferred where only the fundamental S_0_ and A_0_ modes exist to reduce the chances of overlapping of the modes, which, eventually, reduces the complexity during post-processing. For this investigation, the fundamental A_0_ mode is used because of the following reasons:

- The required incidence angle is relatively large when compared to other modes; therefore, the chance that other modes will be generated inside the sample along with A_0_ mode is sufficiently low;
- Due to the shorter wavelength among the fundamental modes, the A_0_ mode will be more sensitive to smaller defects such as disbond and delamination in the composite plate.

Hence, from the dispersion curves, the following values were obtained for the 3.2 mm GFRP plate at 300 kHz excitation frequency: phase velocity of 1424 m/s and group velocity of 1725 m/s; using the phase velocity, the angle of incidence of 14° was calculated using Snell’s law. Additionally, the ultrasound velocity in air at 21 °C was 343.21 m/s, the wavelength in air at 300 kHz was 1.14 mm, and the wavelength of the A_0_ mode in the GFRP plate at 300 kHz was 4.74 mm. In the case of bulk waves, the longitudinal wave velocity was calculated to be 3626 m/s. The wavelength corresponding to the longitudinal velocity at 300 kHz was 12.08 mm and that at 485 kHz was 7.47 mm.

**Figure 5:**
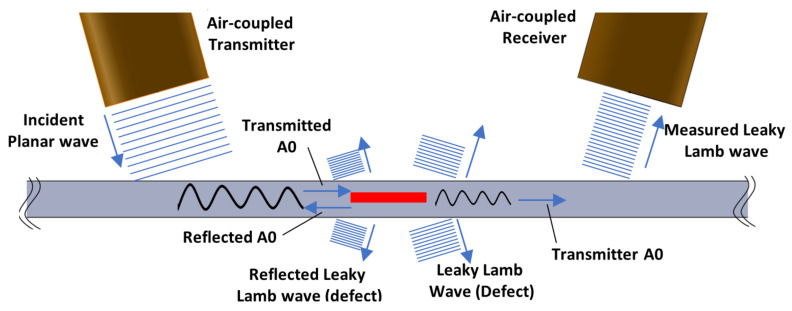
Illustration of different types of ultrasonic waves generated in air-coupled guided wave inspection and their interaction with the internal defect.

The interaction of Lamb waves with the defect can result in mode conversion, scattering, and reflection [38,39]. The guided waves in this study were generated in the composite plate using the air-coupled pitch-catch method. The transducers were placed at the angle of 14° and 100 mm apart from the incident point along the acoustical axes, and they were positioned at 10 mm from the surface of the plate along the acoustical axis. In an air-coupled guided wave setup such as this, the typical ultrasonic waves generated during the interaction with the defect are as illustrated in Figure 5. It is to be noted that this figure represents the loss of energy with respect to the amplitude of the ultrasonic wave but not in terms of the change in frequency or wavelength.

### 3.3. Guided Wave Numerical Simulation

A guided wave numerical simulation was performed to verify the analytical dispersion curves generated for the waveguide and to get insight into the wave propagation, different modes propagating in the composite sample, and the interaction of the guided waves with the internal defects. Such information obtained from the simulation can greatly reduce the time and cost for experimental work. The guided wave model used in this study was created in the finite element software package COMSOL Multiphysics (version 5.5, COMSOL Inc, Burlington, VT, USA), where two different as approaches, i.e., pressure acoustics physics and solid mechanics physics, can be coupled to achieve the air-coupled guided wave phenomenon.

**Figure 6:**
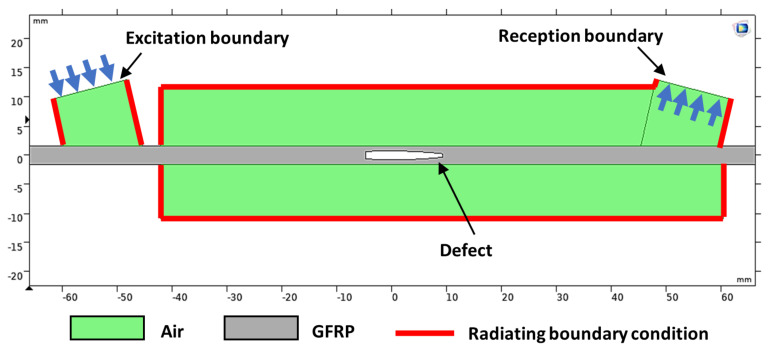
Two-dimensional (2D) air-coupled guided wave simulation model from COMSOL.

The guided wave model is shown in Figure 6 where the air domain is shown in green and the GFRP plate is shown in gray. Here, a simple two-dimensional (2D) model was considered because such a model is sufficient to perform time-of-flight analysis and to get insight into the interaction with the defect. In the created model, the GFRP plate was assumed and defined to be a solid isotropic material with 3.2 mm thickness and 305 mm width, and the air was defined as a fluid medium.

The elastic properties of the GFRP plate were as described in the previous section: *E* = 10.5 GPa, *ν* = 0.36, and *ρ* = 1342 kg/m^3^; the properties used for air at 21 ℃ were as follows: *ρ* = 1.2041 kg/m^3^ and velocity *V_air_* = 343.21 m/s. The boundary condition for the air domain, except for the excitation and reception boundary, was defined as a radiating boundary condition to simulate an infinite medium and to avoid reflections. The excitation and reception domains in the air were modeled in zones with a width of 14 mm, corresponding to the diameter of the 300 kHz flat transducer, and these domains were placed at 14° to generate and receive the A_0_ mode at 300 kHz (Figure 6). The distance between them was 105 mm.

Since the wavelength of the ultrasonic wave in air at 300 kHz was 1.14 mm and was shorter than the wavelength of A_0_ mode in the GFRP plate of 4.74 mm, the mesh size was chosen accordingly. For an optimum solution and effective calculation time, a triangular-shaped mesh with eight elements per wavelength, as suggested in [40], was used. The Courant–Friedrichs–Lewy (*CFL*) condition [41] given by Equation (3) was used to determine the optimum time step for the transient study.

(3) $$CFL=\frac{c\Delta t}{h_{0}},$$

where *c* is the slowest velocity of ultrasonic waves in the model, *h*_0_ is the mesh size for the slowest velocity domain, and Δ*t* is the time step. The *CFL* value of 0.2 was the optimal value for the Lamb wave and, in this study, the slowest velocity was in the air medium. Hence, values *c = V_air_* = 343.21 m/s and *h*_0_ = 0.14 mm yielded a time step of Δ*t* = 83.33 ns. The study was performed for 200 µs, and the results are compared in the upcoming sections.

In this study, the defect was assumed to be a void just to see if there were mode conversion and leaky Lamb waves from the defect. A model with no defect was used for a proper time-of-arrival verification.

### 3.4. Guided Wave Tomography

**Figure 7:**
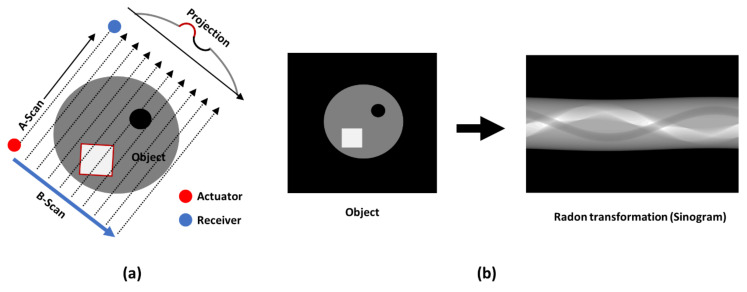
Schema of ultrasonic parallel beam projection (**a**); an example of an object and its sinogram (**b**).

**Figure 8:**
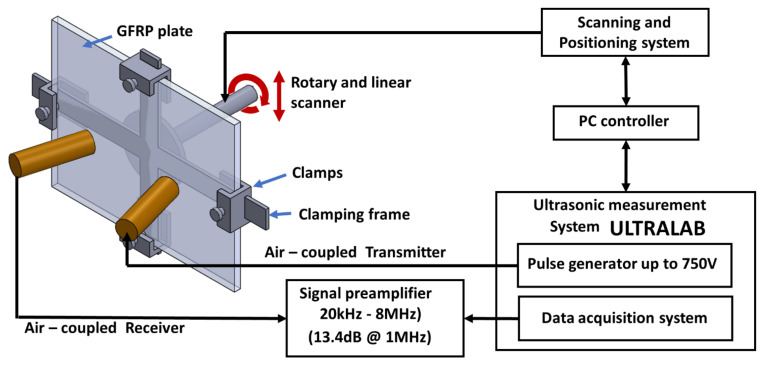
Setup used for guided wave tomography scan.

The guided wave tomography in this study was implemented from a series of parallel projections (B-scans) of the object at different angles, as shown in the schema in Figure 7a. This method of scanning was performed by fixing the transducers and moving the plate with a linear and rotary scanner setup. The linear movement was used to obtain the ultrasonic guided wave B-scan, where the peak amplitude of the envelope of each A-scan resulted in the projection of the silhouette of the impact defect in the GFRP sample. As a result, only the peak amplitude of the envelope of the waveform was considered. The Radon transform is a plot of the peak amplitudes of the scanner data, which represents the integral of the slice of the parallel projection object at different orientations, resembling a sine wave and called a sinogram. An example of a sinogram is shown in Figure 7b. The linear and rotary scanner setup necessary to perform the ultrasonic tomography study was designed and manufactured at the Ultrasound Institute of Kaunas University of Technology, as shown in Figure 8.

Sinograms can be mathematically solved using different techniques [42] such as the traditional filtered back projection (FBP) algorithm, reconstruction algorithm for the probabilistic inspection of damage (RAPID), and difference Hilbert back projection (DHB) algorithm. The RAPID algorithm is not suitable for multi-defect imaging and is best used to visualize faults with fewer transmission and reception points [30]. These limitations can be overcome using the FBP algorithm, where the image precision is better, and it is well suited for multi-defect imaging; the only disadvantage is the visual artefacts, occurring near the scanning edge, where the information is distorted. The DHB algorithm is very good in cases of imaging multiple defects and has fewer distortions near the scanning edges. In this article, the composite impact damage specimen had defects in the center of the scanning area, with the scanning area being bigger than the region of the defect. Therefore, the tomography reconstruction in this article was performed using the FBP algorithm, which was well suited for this investigation. The FBP algorithm is available in mathematical software MATLAB (version 2019a, MathWorks, Natick, MA, USA) under the function *iradon* transform [43].

**Figure 9:**
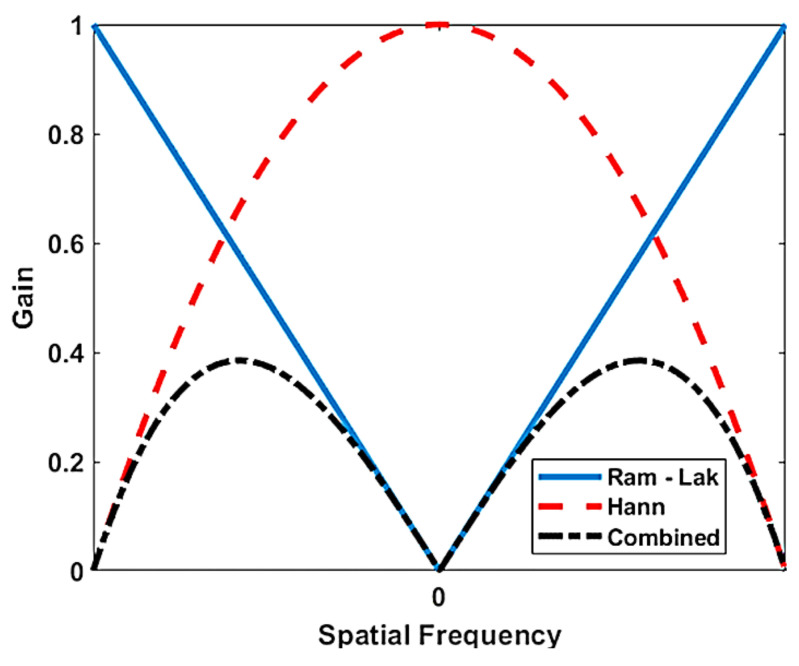
Ram-Lak, Hann, and combined filters in the spatial frequency.

The FBP algorithm can be used with different types of filters such as Hamming, Hann, and cosine. For this investigation, the Hann filter was selected because the Hann window is known for its best performance in the compromise among low image noise, high spatial and frequency resolution, and reduced spectral leakage. The Hann filter is given by

(4) $$Hann=\frac{1}{2}\left(1+\cos\left(\frac{2\pi \omega_{s}}{L}\right)\right),$$

where *ω_s_* is the spatial frequency, and *L* is the window length. This window was combined by multiplying with a Ram-Lak filter, as shown in Figure 9, which was used for the tomographic reconstruction.

## 4. Experimental Results and Discussion

### 4.1. Results of the Air-Coupled through Transmission Method

The through transmission scan was performed with the configuration shown in Figure 3 and, for scanning, the transducers were fixed and the GFRP plate was moved in two directions to obtain the C-scan image of the defect. The C-scan in the case of the 8 J impact defect was performed in an area of 60 × 50 mm^2^, and that in the case of the 12 J impact defect was performed in an area of 60 × 80 mm^2^. The scanning step in both cases was 1 mm in both directions.

**Figure 10:**
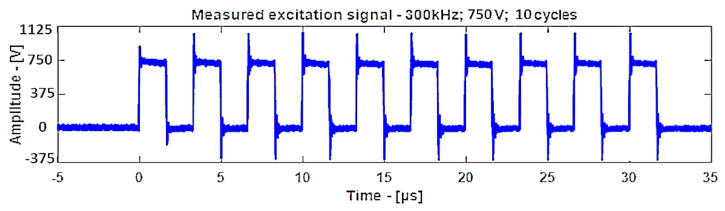
The 300 kHz 10-period rectangular excitation pulse.

The excitation was a 10-period electric rectangular pulse with 750 V amplitude as shown in Figure 10, and the measured signal was averaged 32 times for improving the signal-to-noise ratio. An amplifier with static and dynamic gains of 50 dB and 40 dB was used to amplify the received signal. Similarly, for the C-scan with 485 kHz focused transducers, the other parameters such as the excitation voltage, averaging, number of periods, and amplitude gains remained the same as for the unfocused transducer except for the distance of placement of the transducer.

**Figure 11:**
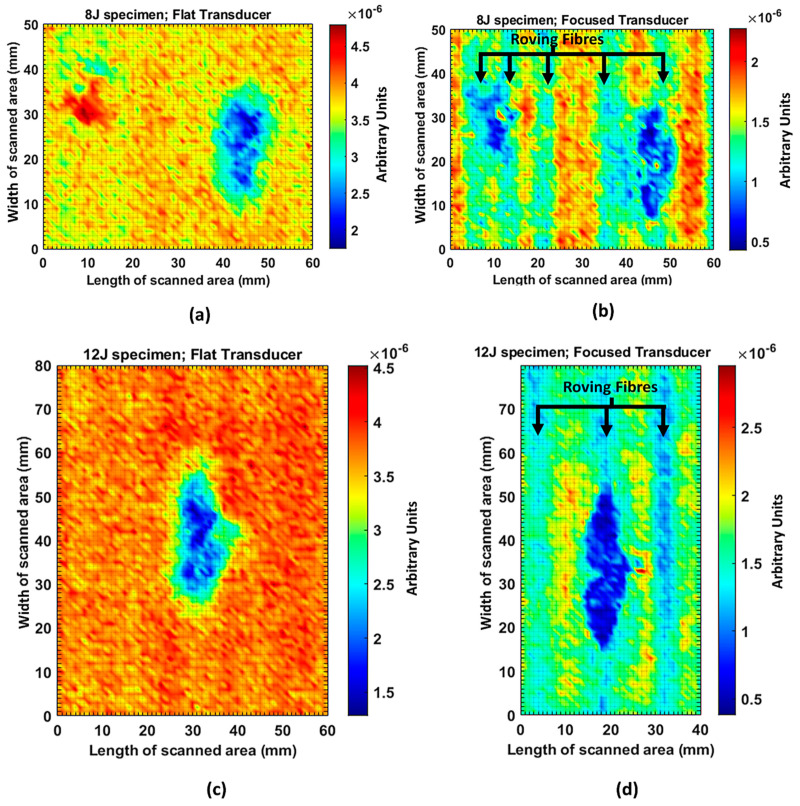
C-scan image for 8 J impact specimen defect using a flat transducer (**a**) and focused transducer (**b**), and for 12 J impact specimen defect using a flat transducer (**c**) and focused transducer (**d**).

The C-scan images shown in Figure 11 are the results obtained from the bulk wave through transmission inspection. The color scale in each image was adjusted to the maximum amplitude of the envelope of each A-scan of the measured signal, so that the scale was always positive. The blue color in the image represents the lowest amplitude of the received signal, which indicates the presence of a defect. However, in the case of one defect in the specimen with the 8 J impact damage, a through-transmitted ultrasonic signal with a high amplitude was observed. The reason for this appearance may have been because of constructive interference or slight variation in distance due to bending on the GFRP plate. However, this effect will be investigated further.

For the specimen with 8 J impact damage (Figure 11a,b), a total of 3721 points were scanned (61 points along the *x*-axis and 51 points along the *y*-axis), and the measurement time taken for the C-scans was 4 h. The results for the specimen with 12 J impact using the flat transducer are shown in Figure 11c, and those for the focused transducer are shown in Figure 11d. For the specimen with 12 J impact damage, a total of 4941 points were scanned (61 points along the *x*-axis and 81 points along the *y*-axis), and the time necessary for the C-scans was 6.6 h. From the results of the C-scan, it is obvious that the results obtained using the focused transducer possessed a higher resolution than the results obtained with the flat transducer. With the focused transducer, even the roving fibers inside the composite plate equally spaced at 15 mm were visible, and the shape of the defect was also much sharper. The defects in the 8 J impact specimen appear to be spread across the neighboring fibers in a direction perpendicular to the roving fiber direction, whereas the defect in the 12 J impact specimen appeared to be concealed within the fiber with a slight protrusion in the middle. The size of the defect was significantly larger running parallel to the roving fiber direction in the case of 12 J impact when compared to the 8 J impact energy.

### 4.2. Results of Guided Wave Simulation

**Figure 12:**
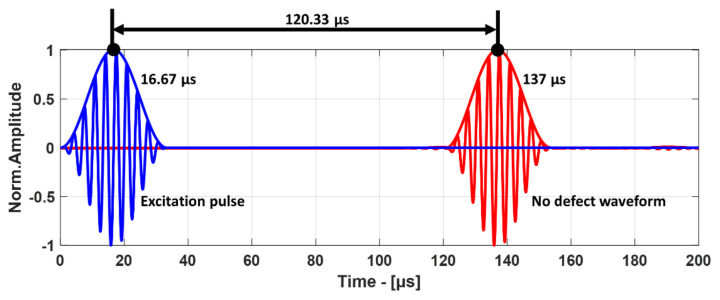
Excitation pulse (blue) and received pulse (red) and their envelopes.

The 10-period Hann windowed sine burst excitation signal with 100 Pa amplitude on the air-coupled transmitter boundary was used to generate the initial plane waves. The function used for excitation signal *V_exe_* is given by Equation (5).

(5) $$V_{exc}=\frac{A}{2}\left(1-\cos\left(\frac{\omega t}{N}\right)\right)\sin\left(\omega t\right)\left(t<\frac{N}{f}\right),$$

where *A* is the pressure amplitude of the excitation signal, *N* is the number of periods, *f* is the frequency of excitation, *t* is the time, and *ω* = 2*πf* is the angular frequency. This plane wave generated the A_0_ mode in the GFRP plate, and resulting leaky Lamb waves were measured in the receiver boundary. The excitation signal and the received signal are shown in Figure 12.

In order to prove that the numerical model was correct, the time of arrival (ToA) measured from the A-scan of the simulation was compared with the analytically calculated ToA obtained using the velocities from dispersion curves. In order to determine the ToA from simulation results, the peak-to-peak method [44] was used, where the Hilbert envelope allowed determining the propagation time of the group of waves with the highest energy. For the analytical calculation, the distance between the transducer and the surface of the GFRP plate was 10 mm for both the transmitter and the receiver, and the speed of sound in air was taken as 343.21 m/s. The distance traveled by the guided wave inside the composite plate was 105 mm with the group velocity of 1752 m/s. Therefore, using the analytical method, the ToA was calculated to be 119.14 µs, which is very close to the ToA of 120.33 µs obtained from the simulation, as shown in Figure 12.

**Figure 13:**
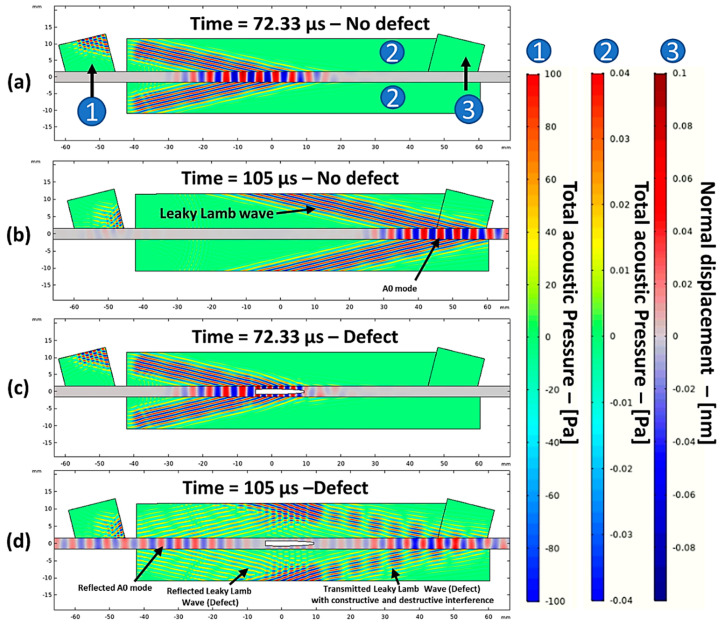
Simulated snapshots in the case of no defect at the time instants of 72.33 µs (**a**) and 105 µs (**b**) and with defect at the same time instants (**c**,**d**). The color scales describe the excitation air domain (1), leaky wave air domains (2), and GFRP domain (3).

**Figure 14:**
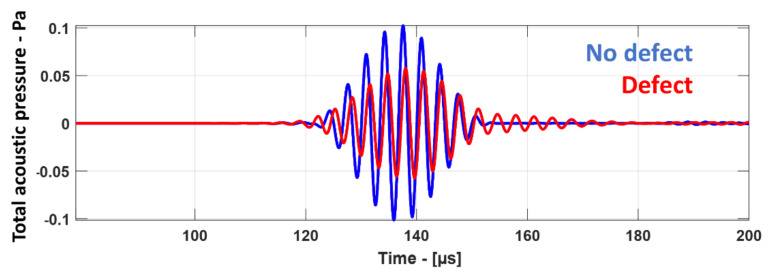
Waveforms measured at the receiver boundary of plate without defect (blue) and with defect (red).

Figure 13 shows the snapshots obtained from the guided wave simulation at different time instances. In the air domain, wave propagation is represented as a total acoustic pressure in Pascals, while, in the GFRP plate (which is a solid domain), it is represented as a normal displacement in millimeters. The plane waves arriving at the reception area (2) had a much lower amplitude than that at the excitation area (1) in Figure 13, because of the transfer losses when interacting with the GFRP plate. Therefore, to be able to see the changes in the acoustic pressure both in excitation and in reception areas in the air domain, they were visualized on different scales, as shown on the right side of Figure 13. From the snapshots, the generation of the A_0_ mode is evident by looking at the displacement profile as shown in Figure 13a,b. The interaction with the defect produced leaky Lamb waves in the air domain and the reflected A_0_ mode in the GFRP plate, as shown in Figure 13c,d. The measured waveform at the receiver boundary is shown in Figure 14, where the signal with the defect showed a lower amplitude with a slight delay when compared to the signal without defect. This change in amplitude and delay indicated the presence of the defect.

### 4.3. Results of Guided Wave B-Scan Technique

**Figure 15:**
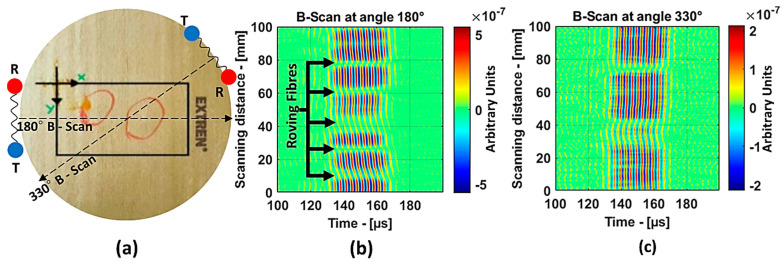
Zoomed in region of the 8 J impact damage specimen indicating the B-scan directions, where T and R represent the transmitter and receiver, respectively (**a**); B-scan results at 180° (**b**) and 330° (**c**).

For the guided wave inspection, the 300 kHz flat transducers were used. The experimental setup of the air-coupled guided wave technique is shown in Figure 8. In this investigation, the guided wave B-scan image was obtained by moving the scanner in the *y*-direction by 1 mm increments for a total distance of 100 mm. The inspection parameters were as follows: excitation voltage, 750 V; number of periods, 10; static and dynamic gains, 50 dB and 40 dB, respectively; 64 times averaging. Each B-scan took about 9 min. The schema of B-scans performed on the 8 J impact specimen at angles 180° and 330° is shown in Figure 15a.

**Figure 16:**
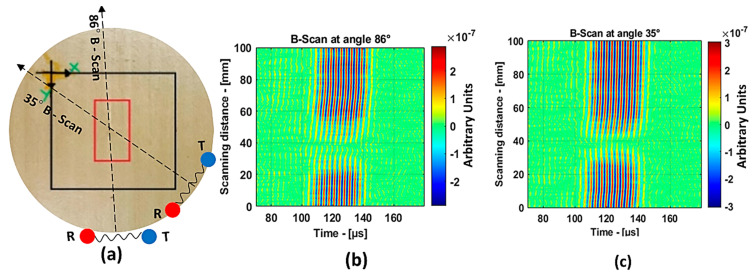
Zoomed-in region of the 12 J impact damage specimen indicating the B-scan directions, where T and R represent the transmitter and receiver, respectively (**a**); B-scan results at 86° (**b**) and 35° (**c**).

Figure 15b represents the B-scan image obtained as the result of moving the transducers perpendicular (180° from the reference) to the roving fiber direction. Approximately, for every 10 mm, a change in the amplitude of the guided waves could be observed, and this is because the energy was lost when traveling inside the roving fibers. Therefore, in this case, obtaining information about the defect was difficult. On the other hand, the result from the 330° angle in Figure 15c shows a clear indication of the presence of two distinct defects in the 8 J specimen. In the case of the 12 J impact specimen, the schema for the B-Scan is shown in Figure 16a. The defect was elongated and, to show large variance in the size of the defect from the B-scan, the results at angles 86° and 35° were chosen, as shown in Figure 16b,c, respectively.

The B-scan images show a clear indication that in the direction of 86°, it was possible to detect the long side of the 12 J impact damage, and, in the direction of 35°, it was possible to detect the short side of the 12 J impact damage. Furthermore, the presented B-scans show that the time of flight was approximately 130 µs, which is close to the simulation results. This proves the validity of the used numerical model.

### 4.4. Results of Guided Wave Tomography

**Figure 17:**
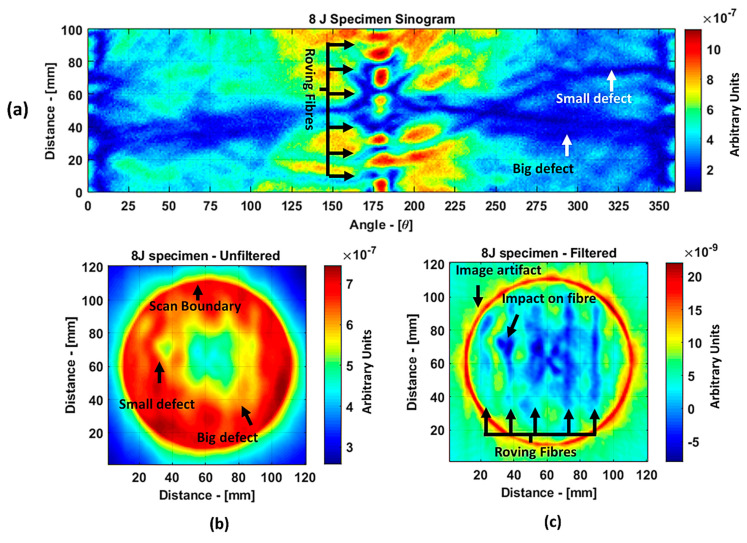
Tomography reconstruction of 8 J impact damage: sinogram (**a**); unfiltered reconstruction (**b**); filtered reconstruction (**c**).

Guided wave tomography is the extended version of the guided wave B-scan method, where the B-scans were obtained for 360° with the angular step of 1°. Thus, to obtain data for tomographic reconstruction with 1° step, it took approximately 2.5 days. As discussed before, the sinogram of the structure was obtained by recording the peak value of the envelope of each A-scan in the B-scan. The sinogram for the 8 J impact defect is shown in Figure 17a, where a clear indication of curves with low-amplitude values representing the two defects (blue color) can be observed. It is interesting to see the roving fibers visible at angles 0°, 180°, and 360°, where the B-scan direction was parallel to the direction of the roving fiber inside the specimen. The roving fibers were at the positions where the amplitude was lowest (blue color) at the mentioned angles.

The reconstruction algorithm was performed using the inverse Radon function that is available in the MATLAB image processing library. The reconstruction was performed without frequency domain filtering and using the frequency domain filter, as shown in Figure 17b,c, respectively. The unfiltered reconstructed image was obtained using the linear interpolation, while the filtered image is obtained using the shape-preserving interpolation with the Hann filter. In the reconstructed images, an image artefact due to the boundary of the scan was observed, as mentioned in [30]. However, these edge artefacts did not influence the quality of the reconstruction because of the large scan area.

**Figure 18:**
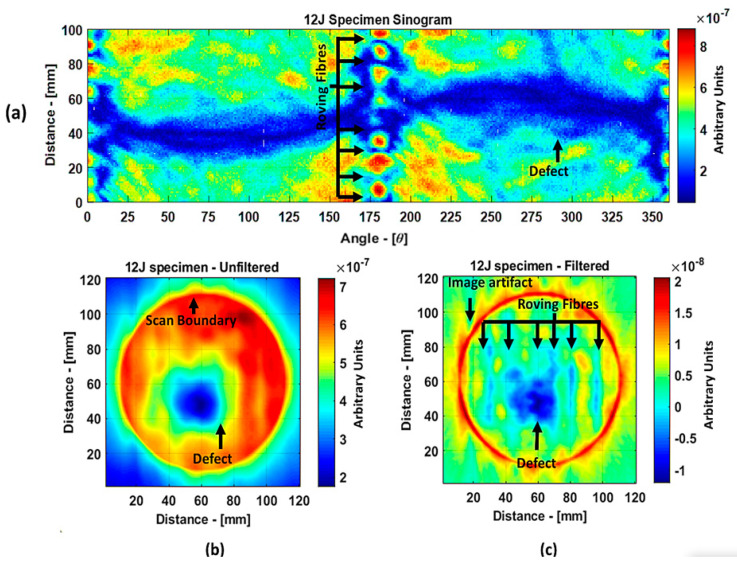
Tomography reconstruction of 12 J impact damage: sinogram (**a**); unfiltered reconstruction (**b**); filtered reconstruction (**c**).

The reason why the small defect was not visible from angles 0° to 125° is that, when starting the scan, the big defect shadowed the smaller ones on the path of the guided wave such that no information about the small defect was obtained through the projections. In the unfiltered reconstructed image of the 8 J damage specimen, the small and big defects can be observed, while, in the filtered reconstruction image, the damage on the roving fiber and its deformation was visible in place of the small defect. The big defect’s size and shape were not evident when compared to the unfiltered image. In the case of the 12 J impact specimen, the sinogram and the unfiltered and filtered images are shown in Figure 18a–c. The 12 J impact damage shown in the sinogram (Figure 18a) is clear because there was only one defect in this specimen. In this specimen, the roving fibers were visible (Figure 18c) as in the previous specimen.

The reconstructed image of the damage shows that the defect was almost circular in shape and that the defect was spread across two roving fibers (Figure 18c). In the case of guided waves in A_0_ mode, they traveled inside the sample with a high normal displacement energy. Therefore, even the slightest damage would show a significant change in amplitude. Hence, there is a possibility that the extent of the 12 J impact defect was more parallel to the waveguide.

## 5. Defect Sizing and Discussion

The three different types of air-coupled methods, namely, the through transmission technique, the guided wave technique, and the guided wave tomographic reconstruction technique, for detection of impact-type defects in the pultruded GFRP samples with 3.2 mm thickness were compared. The study was performed using the air-coupled unfocused transducers at 300 kHz and the focused composite transducers at 485 kHz. The results from the 8 J impact specimen and 12 J impact specimen were evaluated using the −6 dB technique, and the results are presented in Table 2 and Table 3, respectively.

According to the investigation and results, the following observations were made, which are also compared and summarized in Table 4.

- The results obtained using the air-coupled through transmission technique with the focused transducers showed detailed information about the impact defects in the GFRP samples, and even the internal fibers could be observed. However, in the case of the flat transducers, the internal fibers could not be identified. The defects appeared larger than in the results from the focused transducers.

This is mainly because, in the case of the flat transducers, the signals of the received waves were integrated throughout the 14 mm diameter circular surface of the transducer, while, in the case of the focused transducers, a focal resolution of up to 1.5 mm was possible. Another reason is that the lower frequency of excitation of 300 kHz resulted in a longer wavelength and a lower resolution. For each C-scan, a total of 3.3 h was consumed. The possibility of in situ inspection is low because there is a requirement for two-side access to the structure.

- For the air-coupled guided wave investigation, the defect size could be measured, but the information about the shape of the defect could not be obtained. The size of the defect determined was relatively close to the size determined by the through transmission technique. There is a possibility of in situ inspection because it is faster (only 10 min was taken for each scan) and has the advantage of allowing one-sided inspection of the sample. The setup is quite simple.
- Using the tomographic reconstruction, the problem of the guided wave technique, where the shape of a defect was impossible to be determined, could be tackled. The guided wave tomography results showed that, with the filtered tomographic reconstruction, the location of the defect, as well as the internal roving fiber structures, with the same 300 kHz flat transducers could be determined. This result is quite impressive because this technique opens the possibility of imaging the internal structure of a sample without using higher-frequency transducers.

There was a drawback whereby the shape of the defect reconstructed was not as close to the shape of the defect when compared to the results obtained using the through transmission technique. This is mainly due to the fact that, in the case of the through transmission technique, the incident ultrasonic wave was perpendicular to the plate and, therefore, was less susceptible to dispersion due to the geometry of the sample. In the guided wave technique, the guided waves were influenced by the spatial variations of the internal structure of the guided medium. Given the random fiber orientation of the CFM, this kind of effect was inevitable. Another drawback was the time consumption for each scan. It took about 2.5 days for a full tomographic scan. Nevertheless, if the inspection time is not a constraint, for example, in the case of the investigation of fatal events, this technique can be quite useful.

## 6. Conclusions

Three different types of air-coupled testing techniques (through transmission, guided waves, and guided wave tomography) were compared, and the following conclusions were made:

- The air-coupled through transmission technique can provide the highest spatial resolution of defects when using focused transducers at higher frequency (e.g., 485 kHz).
- The guided wave technique is recommended due to its ability of single-side inspection of the sample. In most cases of assembled composites structures, two-side access is not possible. In the case of the guided wave inspection, a far simpler setup can easily be used for in situ NDT.
- Numerical simulations can be of significant use to understand the effects of guided wave propagation in composite samples, mode conversions, and interaction of the guided waves with internal defects. Such simulations and results obtained by them were used to select the appropriate frequency of inspection and configuration of the experimental setup. Since multiple modes can exist at the same time in the guided wave technique, only frequencies lower than 400 kHz can be used for inspection for such specific GFRP plates.
- The through transmission technique can consume more time for inspection as the area of scan increases, while the guided wave technique can be used to inspect large areas in a single scan.
- The detectability of defects using the through transmission technique and the guided wave technique can vary because, in the first technique, the incident ultrasonic waves are perpendicular to the structure, whereas, in the second technique, the ultrasonic waves propagate parallel to the surfaces of the sample. Therefore, the orientation of a defect can influence detectability. In the case of impact defects, both techniques show good results.
- Even at lower frequencies (e.g., 300 kHz), much more detail regarding the internal structure of the GFRP plate can be revealed in the case of the guided wave tomography technique compared to the through transmission method.

## Figures and Tables

**Table 1:** Properties of materials used in GFRP pultruded composite.

Material	Young Module (GPa) E	Poisson Ratio (−) v	Density (kg·m^−3^) ρ
Vinyl ester	3.59	0.38	1200
E-Glass	72.35	0.22	2620

**Table 2:** Defect sizing of 8 J impact specimen with −6 dB method.

	8 J Impact Defect 1	Defect Size (−6 dB)	8 J Impact Defect 2	Defect Size (−6 dB)
Original Specimen	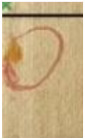	5 × 5 mm^2^	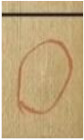	10 × 15 mm^2^
300 kHz Unfocused through Transmission C-Scan	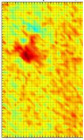	12 × 6 mm^2^	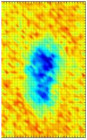	21 × 16 mm^2^
485 kHz Focused through Transmission C-Scan	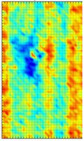	18 × 11 mm^2^	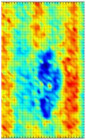	26 × 13 mm^2^
300 kHz Guided Wave B-Scan θ = 330°	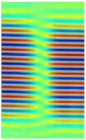	10 mm^2^	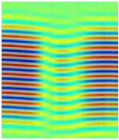	22 mm^2^
300 kHz Guided Wave Tomography Unfiltered	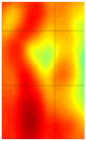	8 × 8 mm^2^	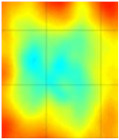	38 × 34 mm^2^
300 kHz Guided Wave Tomography Filtered	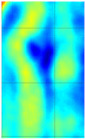	11 × 12 mm^2^	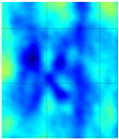	37 × 32 mm^2^

**Table 3:** Defect sizing of 12 J impact specimen with −6 dB method.

	12 J Impact Defect	Defect Size (−6 dB)
Original Specimen	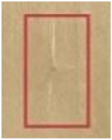	25 × 13 mm^2^
300 kHz Unfocused through Transmission C-Scan	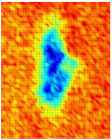	27 × 12 mm^2^
485 kHz Focused through Transmission C-Scan	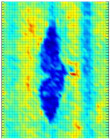	33 × 10 mm^2^
300 kHz Guided Wave B-Scan	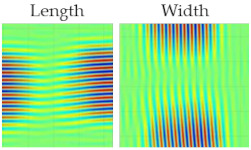	θ = 86° 10 mm (length) θ = 35° 22 mm (width)
300 kHz Guided Wave Tomography Unfiltered	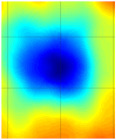	22 × 34 mm^2^
300 kHz Guided Wave Tomography Filtered	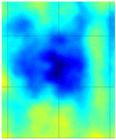	36 × 32 mm^2^

**Table 4:** Comparison of different air-coupled techniques for GFRP pultruded sample.

	Air-Coupled through Transmission		Air-Coupled Guided Waves	
	Flat Transducersat 300 kHz	Focused Transducersat 485 kHz	Guided Wave B-Scan	Guided Wave Tomography
Capabilities and advantages	Contactless coupling; approximate representation of defect; easy transducer placement.	Contactless coupling; focused beams; accurate representation of defect size.	The ability of fast inspection; single-sided inspection; simple and portable experimental setup.	Ability to see internal structural details even at lower frequency; a large area of coverage.
Limitations	The requirement for two-side access to the structure; nonportable experimental setup.	The requirement for two-side access to the structure; difficult to focus the transducers; nonportable experimental setup.	Low signal-to-noise ratio (SNR) which can be improved by averaging and adjusting the orientation angle of the transducers.	The same limitations as of the guided wave technique and complex experimental setup.
Resolution of defect sizing	Has a wavelength of 12 mm; however, for plate-like structures, defects can be identified as small as 4 mm, as seen from 8 J specimen results.	Even though the wavelength at 485 kHz is 7.5 mm, defects as small as 1 mm can be identified due to smaller focal diameter.	Good for detection of defects bigger than 5 mm due to the wavelength being 4.7 mm at 300 kHz.	Good for defects with a size bigger than 5 mm due to the wavelength being 4.7 mm at 300 kHz.
Time consumption	3.3 h for the area of 60 × 50 mm^2^.	3.3 h for the area of 60 × 50 mm^2^.	Practically requires a few minutes for 100 mm scan.	The most time-consuming scan takes about 2.5 days for a 100 × 100 mm^2^ area scan.
Recommendation for in situ inspection	Less favorable due to low resolution.	A less favorable but better choice than unfocused transducers.	More favorable for fast and coarse scanning due to one-sided inspection.	Recommended if time is not a constraint.

## Data Availability

The data presented in this study are available on request from the corresponding author. The data are not publicly available due to usage of developed algorithms.
